# Changes in V1 orientation tuning when blocking astrocytic glutamate transporters: models for extra- and intrasynaptic mechanisms

**DOI:** 10.1186/1471-2202-14-S1-P298

**Published:** 2013-07-08

**Authors:** Konstantin Mergenthaler, Dipanjan Roy, Jeremy Petravicz, Mriganka Sur, Klaus Obermayer

**Affiliations:** 1 Neural Information Processing, School of Computer Science and Electrical Engineering and Bernstein Center for Computational Neurocience, Technische Universität Berlin, Germany; 2 Department of Brain and Cognitive Sciences, Piclower Institut for Learning and Memory, Massachusetts Institute of Technology, Cambridge, Massachusetts, USA

## 

Several recent studies reveal a close interplay of neurons and astrocytes in information processing [1]. Astrocytes affect neural transmission by their control of glutamate concentrations by glutamate transporters [2] in direct vicinity to the synaptic cleft as well as extrasynaptically. An in-vivo study in primary visual cortex of ferrets [3] demonstrated the impact of astrocytic glutamate transport on neuronal response by blocking glutamate uptake pharmacologically with TBOA. A severe effect on neuronal orientation tuning curves to a full-field stationary grating, tilted every second by additional 10 degrees, was observed. While glutamate transporters were blocked tuning curves were broadened (HWHM increased from 28 deg. to 39 deg.) and response at preferred orientation was enhanced. However, it is unclear if the change in response originates from a prolongation of synaptic glutamate clearance or locally increased ambient glutamate concentration. Here we investigate in a computational model of ferret V1 how the intra- and extrasynaptic mechanisms affect orientation tuning.

We implemented both mechanisms in a highly recurrent single layer 2d map model based on [4]. Neurons are placed on a 50 × 50 grid and connected to their neighbors by randomly drawn connections from a Gaussian. On every grid point an excitatory neuron is placed, additionally every third grid point is occupied by an inhibitory neuron. Peak conductivities for the four types of connections are set to be in the recurrent regime, exceeding the conductivity of the additional afferent input. The afferent input specific to each grid location induces a pinwheel-domain organization of neurons in the network. For the implementation of the synaptic mechanism the glutamate concentration follows a bi-exponential description with a decay time prolonged if glutamate uptake is reduced [2]. Synaptic glutamate then activates complex kinetic NMDA and AMPA-receptors [5]. The synaptic mechanism can however have a stronger influence on excitatory-to-excitatory or excitatory-to-inhibitory connections, as the different synapse geometries also affects glutamate clearance. Extrasynaptically ambient glutamate provides a constant NMDA-receptor mediated somatic current [6]. Here different sensitivities (different NMDA-receptor densities) of excitatory and inhibitory neurons to ambient glutamate may affects tuning. For both mechanism possible parameter combinations are assessed in a grid search.

We observed that a selective increase in modalities towards inhibitory neurons even leads to a sharpening of orientation tuning. While selective enhancement in the modalities towards excitatory neurons leads first to a drop in orientation tuning and very fast for further increase to pathological firing rates. For both mechanisms the closest fit in orientation tuning (HWHM) to the experimental observation with TBOA was found for a stronger effect on excitatory neurons along with a simultaneous but weaker effect on the inhibitory population. While both models can explain the current data, they, however, provide different predictions for sub-threshold properties and for neurons close to pinwheels or domain centers.
